# Expression of C-terminal ALK, RET, or ROS1 in lung cancer cells with or without fusion

**DOI:** 10.1186/s12885-019-5527-2

**Published:** 2019-04-03

**Authors:** Koh Furugaki, Marie Mochizuki, Mirei Kohno, Sei Shu, Naoki Harada, Yasushi Yoshimura

**Affiliations:** Product Research Department, Kamakura Research Laboratories, Chugai Pharmaceutical Co., Ltd., 200 Kajiwara, Kamakura, Kanagawa 247-8530 Japan

**Keywords:** EML4, ALK, RET, ROS1, Lung cancer, Alectinib, Immunohistochemistry, Rearrangement, Fusion

## Abstract

**Background:**

Genetic alterations, including mutation of epidermal growth factor receptor or v-Ki-ras2 kirsten rat sarcoma viral oncogene homolog and fusion of anaplastic lymphoma kinase (*ALK*), RET proto-oncogene (*RET*), or v-ros UR2 sarcoma virus oncogene homolog 1 (*ROS1*), occur in non-small cell lung cancers, and these oncogenic drivers are important biomarkers for targeted therapies. A useful technique to screen for these fusions is the detection of native carboxy-terminal (C-terminal) protein by immunohistochemistry; however, the effects of other genetic alterations on C-terminal expression is not fully understood. In this study, we evaluated whether C-terminal expression is specifically elevated by fusion with or without typical genetic alterations of lung cancer.

**Methods:**

In 37 human lung cancer cell lines and four tissue specimens, protein and mRNA levels were measured by capillary western blotting and reverse transcription–PCR, respectively.

**Results:**

Compared with the median of all 37 cell lines, mRNA levels at the C-terminus of all five of the fusion-positive cell lines tested (three *ALK*, one *RET*, and one *ROS1*) were elevated at least 2000-, 300-, or 2000-fold, respectively, and high C-terminal protein expression was detected. In an *ALK* fusion–positive tissue specimen, the mRNA and protein levels of C-terminal ALK were also markedly elevated. Meanwhile, in one of 36 *RET* fusion–negative cell lines, *RET* mRNA levels at the C-terminus were elevated at least 500-fold compared with the median of all 37 cell lines, and high C-terminal protein expression was detected despite the absence of *RET* fusion.

**Conclusions:**

This study of 37 cell lines and four tissue specimens shows the detection of C-terminal ALK or ROS1 proteins could be a comprehensive method to determine *ALK* or *ROS1* fusion, whereas not only the detection of C-terminal RET protein but also other methods would be needed to determine *RET* fusion.

**Electronic supplementary material:**

The online version of this article (10.1186/s12885-019-5527-2) contains supplementary material, which is available to authorized users.

## Background

Molecular subsets of non-small cell lung cancer (NSCLC) have been defined by various types of driver gene mutations involving epidermal growth factor receptor (*EGFR*), v-Ki-ras2 Kirsten rat sarcoma viral oncogene homolog (*KRAS*), and anaplastic lymphoma kinase (ALK) gene fusion. Specific tyrosine kinase inhibitors (TKIs), such as EGFR-TKIs (erlotinib and afatinib) and ALK-TKIs (crizotinib and alectinib), that inhibit the oncogenic activity of these genes have been developed and approved [1, 2]. A key issue in identifying patients that would be suitable for the targeted agents is precisely identifying the presence or absence of the driver gene mutations in a molecular diagnosis of the lung cancer. In approximately 5% of NSCLC, the rearrangement of the amino-terminal (N-terminal) region of echinoderm microtubule associated protein like 4 (*EML4*) with the carboxy-terminal (C-terminal) region of ALK occurs by inversion within the short arm of chromosome 2 [3]. In cancer cells with *EML4-ALK*, the transcription of the C-terminal region of ALK depends on the promoter activity of the fusion partner, *EML4*, which is a housekeeping gene for the stabilization of microtubules during mitosis, by which the C-terminal ALK protein level also becomes elevated [4, 5].

To detect *ALK* fusion, there are three different techniques: fluorescence in situ hybridization (FISH), immunohistochemistry (IHC) and reverse transcription–PCR (RT-PCR) are available for the detection of *ALK* fusion [3]. Although each test has some advantages and disadvantages, IHC is more useful as a routine screening method in clinical settings because of cost effectiveness and technical ease [3]. The ALK IHC method determines whether tumor cells are harboring an *ALK* fusion using an antibody directed to the C-terminal ALK protein, but unlike FISH tests, it has been reported to show not only positive results in patients with *ALK* fusion–positive cancer but also false-negative errors in some patients who actually have *ALK* fusion–positive cancer [3, 6–8]. In addition to *ALK* fusion, RET proto-oncogene (*RET*) or v-ros UR2 sarcoma virus oncogene homolog 1 (*ROS1*) are rearranged in approximately 1% of NSCLC. In consequence, RET-TKIs (such as alectinib or vandetanib) and ROS1-TKIs (such as crizotinib or lorlatinib) are under development for fusion-positive NSCLC patients, and precise diagnostic methods for these fusions are needed [2, 9–11].

In this study, we verified the reliability of IHC methods that target ALK, RET, and ROS1 C-terminal protein as diagnostic tools for lung cancer by investigating whether the expression at the C-terminal region is elevated in each of the fusion-positive lung cancer cells compared with that in fusion-negative cells.

## Methods

### Lung cancer cell lines

The histology, driver gene mutation, culture medium, and supplier of the 37 human lung cancer cell lines are listed in Table 1 [12–14]. Cells were purchased from the American Type Culture Collection (ATCC; Manassas, VA), Korean Cell Line Bank (KCLB; Seoul, South Korea), Riken Bioresource Center (Ibaraki, Japan), Health Science Research Resources Bank (HSRRB; Osaka, Japan), National Cancer Institute (NCI; Bethesda, MD), Immuno-Biological Laboratories (IBL; Gunma, Japan), and Leibniz Institute DSMZ-German Collection (DSMZ; Braunschweig, Germany) and were maintained using RPMI1640 (Sigma-Aldrich (SIGMA); St. Louis, MO), minimum essential medium (MEM; SIGMA), McCoy’s 5A (Thermo Fisher Scientific; Waltham, MA), or Ham’s nutrient mixture F12 (HAMF12; SIGMA) supplemented with fetal bovine serum (FBS; Japan Bio Serum, Hiroshima, Japan) at 37 °C in 5% CO_2_. Authentication of all cell lines was conducted by DNA fingerprinting with short tandem repeat profiling using the Powerplex 16 HS system (Promega; Madison, WI).

**Table 1:** 37 lung cancer cell lines

N	Cell line	Histology	Driver gene mutation	Supplier	Catalogue number	Culture medium
1	NCI-H2228	AD	EML4-ALK^1^	ATCC	CRL-5935	10%FBS-RPMI1640
2	SNU-2535	NS	EML4-ALK, G1269A^2^	KCLB	02535	10%FBS-RPMI1640
3	SNU-2292	AD	EML4-ALK^3^	KCLB	02292	10%FBS-RPMI1640
4	HCC827	AD	EGFR E746_A750 DL^1^	ATCC	CRL-2868	10%FBS-RPMI1640
5	PC-9	AD	EGFR E746_A750 DL^1^	IBL	37,012	10%FBS-RPMI1640
6	B901L	AD	EGFR E746_A750 DL^1^	RIKEN	RCB3530	10%FBS-RPMI1640
7	HCC4006	AD	EGFR L747_E749 DL, A750P^4^	ATCC	CRL-2871	10%FBS-RPMI1640
8	HCC2935	AD	EGFR E746_T751 DL, S752I^4^	ATCC	CRL-2869	10%FBS-RPMI1640
9	PC-3	AD	EGFR L747_A750 DL^1^	HSRRB	JCRB0077	10%FBS-MEM
10	NCI-H1650	AD	EGFR E746_A750 DL^1^	ATCC	CRL-5883	10%FBS-RPMI1640
11	II-18	AD	EGFR L858R^1^	RIKEN	RCB2093	10%FBS-RPMI1640
12	NCI-H1975	AD	EGFR L858R, T790M^1^	ATCC	CRL-5908	10%FBS-RPMI1640
13	NCI-H820	AD	EGFR E746_T751 DL, T790M^5^	ATCC	HTB-181	10%FBS-RPMI1640
14	Calu-1	SQ	KRAS G12C^1^	ATCC	HTB-54	10%FBS-McCoy’s 5A
15	NCI-H358	AD	KRAS G12C^1^	ATCC	CRL-5807	10%FBS-RPMI1640
16	HOP-62	AD	KRAS G12C^1^	NCI	502,467	10%FBS-RPMI1640
17	NCI-H2122	AD	KRAS G12C^1^	ATCC	CRL-5985	10%FBS-RPMI1640
18	Calu-6	AC	KRAS Q61K^1^	ATCC	HTB-56	10%FBS-MEM
19	NCI-H460	LC	KRAS Q61H, PIK3CA E545K^1^	ATCC	HTB-177	10%FBS-RPMI1640
20	NCI-H596	AS	PIK3CA E545K^1^	ATCC	HTB-178	10%FBS-RPMI1640
21	NCI-H1781	AD	ERBB2 G776VC^1^	ATCC	CRL-5894	10%FBS-RPMI1640
22	NCI-H1755	AD	BRAF G469A^1^	ATCC	CRL-5892	10%FBS-RPMI1640
23	LC-2/ad	AD	CCDC6-RET^6^	RIKEN	RCB0440	15%FBS-HAMF12
24	HCC78	AD	SLC34A2-ROS1^1^	DSMZ	ACC563	10%FBS-RPMI1640
25	NCI-H2347	AD	NRAS Q61R^1^	ATCC	CRL-5942	10%FBS-RPMI1640
26	NCI-H1993	AD	MET amplification^1^	ATCC	CRL-5909	10%FBS-RPMI1640
27	NCI-H1568	AD	ND^1^	ATCC	CRL-5876	10%FBS-RPMI1640
28	NCI-H522	AD	ND^1^	ATCC	CRL-5810	10%FBS-RPMI1640
29	NCI-H838	AD	ND^1^	ATCC	CRL-5844	10%FBS-RPMI1640
30	A529L	AS	ND^1^	RIKEN	RCB2817	10%FBS-RPMI1640
31	NCI-H1703	AD	ND^1^	ATCC	CRL-5889	10%FBS-RPMI1640
32	NCI-H520	SQ	ND^1^	ATCC	HTB-182	10%FBS-RPMI1640
33	NCI-H2170	SQ	ND^1^	ATCC	CRL-5928	10%FBS-RPMI1640
34	NCI-H226	SQ	ND^1^	ATCC	CRL-5826	10%FBS-RPMI1640
35	SK-MES-1	SQ	ND^1^	ATCC	HTB-58	10%FBS-MEM
36	NCI-H1915	LC	ND^1^	ATCC	CRL-5904	10%FBS-RPMI1640
37	NCI-H292	MC	ND^1^	ATCC	CRL-1848	10%FBS-RPMI1640

*AD* Adenocarcinoma, *NS* Non-small cell carcinoma, *SQ* Squamous carcinoma, *AC* Anaplastic carcinoma, *LC* Large cell carcinoma, *AS* Adenosquamous carcinoma, *MC* Mucoepidermoid carcinoma, *DL* Deletion, *ND* Not detected. Mutations were referred from ^1^ COSMIC cell database, ^2^ Yoshimura Y. et al., ^3^ Additional file 5: Figure S1a,^4^ ATCC’s datasheet, ^5^ Shimamura T. et al., ^6^ Matsubara D. et al.

### Lung cancer tissue specimens

Four frozen lung cancer tissue specimens with paired non-tumor normal adjacent tissue (NAT) were commercially obtained from BioreclamationIVT (Hicksville, NY), and kept at − 80 °C until analysis. The specimens and their clinical and pathological features are listed in Table 2. All studies were ethically reviewed and approved by the ethical review committee at Chugai Pharmaceutical Co., Ltd. The committee is independent from the commercial aspect of the company by involving third-party members.

**Table 2:** Four tissue specimens with lung cancer

N	Specimen ID	Driver gene mutation	Histology	Distance of NAT from tumor
1	ILS31007	*EML4-ALK*	AD	3 cm
2	ILS33976	*EGFR* exon 19 deletion	AD	3 cm
3	ILS31624	*KRAS* G12C	AD	6 cm
4	ILS31026	ND	SQ	5 cm

Each driver gene mutation was determined by Cancer Personalized Profiling by Deep Sequencing or the cobas 4800 mutation test at BioreclamationIVT. *ND* Not detected, *AD* Adenocarcinoma, *SQ* Squamous carcinoma, *NAT* Normal adjacent tissue

### RT-PCR assay

RNA was obtained from the cells using a SV total RNA isolation system (Promega), and the cDNA was synthesized using a PrimeScript RT reagent kit (Takara Bio; Shiga, Japan). RT-PCR was performed using the LightCycler 480 system (Roche Diagnostics; Basel, Switzerland) and Taqman probes (Thermo Fisher Scientific) (Additional file 1: Tables S1 and Additional file 2: Table S2). The value of target mRNA expression normalized by *GAPDH* mRNA was calculated from the crossing point PCR-cycle of each mRNA using the LightCycler 480 software.

### Western blotting assay

Western blotting was performed by the capillary electrophoresis–based protein analysis system, Sally Sue (ProteinSimple; Santa Clara, CA), as described previously [15]. The same amount of protein lysate was loaded in each analysis. The antibodies were shown in Additional file 3: Table S3, and we determined which terminus of protein is recognized by the antibody with reference to the supplier’s datasheet and NCBI reference sequence database (RefSeq) (Additional file 3: Table S3 and Additional file 4: Table S4).

## Results

### Expression of EML4 or ALK in cancer cell lines with or without *ALK* fusion

**Fig. 1:**
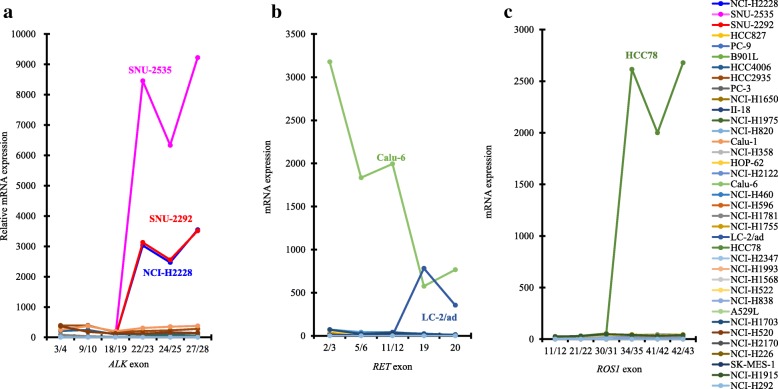
RT-PCR analysis of *ALK*, *RET*, or *ROS1* mRNA in 37 cancer cell lines. The mRNA expression of *ALK* (**a**), *RET* (**b**), or *ROS1* (**c**) was determined by RT-PCR using the aqman probes (Additional file 2: Tables S2). Exons of each probe are shown on the X-axis. The relative mRNA expression was calculated as the ratio of the normalized value with *GAPDH* mRNA to the median of that in 37 cell lines

**Fig. 2:**
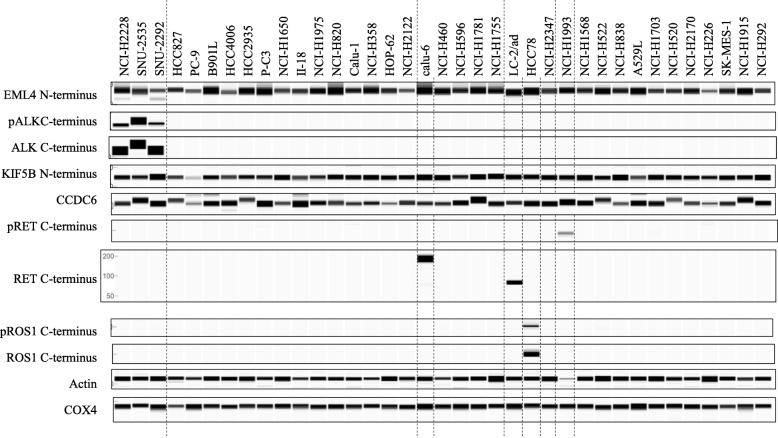
Western blotting analysis in 37 lung cancer cell lines. The protein expression of ALK, RET, ROS1, KIF5B, or CCDC6 and phosphorylation of ALK, RET, or ROS1 in 37 cell lines was determined by western blotting using the antibodies (Additional file 3: Tables S3). Both actin and COX4 were used as loading controls

To examine ALK expression at the C-terminus in lung cancer cells with or without *ALK* fusion, we used 37 lung cancer cell lines (Table 1) that harbor already-known driver mutations, including *ALK* fusion and wild-type, to mimic the populations of patients with lung cancer as shown in Korpanty G.J. et al. [2]. Both the NCI-H2228 and SNU-2292 cell lines had *EML4-ALK* variant 3a, and the SNU-2535 cell line had *EML4-ALK* variant 1 (Additional file 5: Figure S1a and Additional file 6: Figure S2). All ALK-TKIs (alectinib, crizotinib, and ceritinib) inhibited the cell growth of all *ALK* fusion–positive cell lines (IC_50_: < 500 nM) (Additional file 7: Table S5). However, the EGFR-TKI, erlotinib, did not inhibit the cell growth of *ALK* fusion–positive cell lines (IC_50_: > 1000 nM) (Additional file 7: Table S5), indicating that growth of these *ALK* fusion–positive cell lines strongly depends on the signal from ALK kinase. Moreover, in all *ALK* fusion–positive cell lines, mRNA levels of *ALK* at the C-terminal region, which is backward from the breakpoint at exon 20 in *ALK* rearrangement [4], were elevated by at least 2000-fold more than the median of all 37 cell lines, and high protein expression at the C-terminus was detected (Figs. 1a and 2). None of the *ALK* fusion–negative cell lines expressed any mRNA or C-terminal ALK protein. Meanwhile, the protein expression of N-terminal EML4 was detected in each of the 37 cell lines, independent of *ALK* fusion status (Fig. 2 and Additional file 8: Figure S4a).

### Expression of EML4 or ALK in tissues with or without *ALK* fusion

**Fig. 3:**
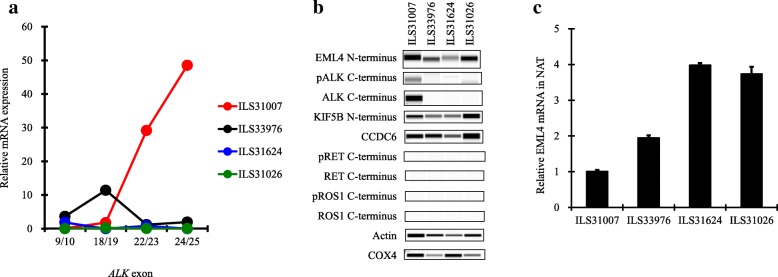
RT-PCR and western blotting analysis in four tissue specimens. The mRNA expression of *ALK* in the four tumor tissue specimens shown in Table 2 was determined by RT-PCR using the Taqman probes (Additional file 2: Tables S2). Exons of each probe are shown on the X-axis. The relative mRNA expression was calculated as the ratio of the normalized value with *GAPDH* mRNA to the median of that in the four tissue specimens (**a**). The protein expression of ALK, RET, ROS1, KIF5B, or CCDC6 and phosphorylation of ALK, RET, or ROS1 in the tumor tissue specimens were determined by western blotting using the antibodies (Additional file 3: Tables S3) (**b**). The relative *EML4* mRNA expression at exon 1/2 in NAT specimens was calculated as the ratio of the normalized values with *GAPDH* mRNA to that in tumor tissue specimens (**c**). Each bar represents the mean + SD (*n* = 3)

Next, we tested four lung cancer tissue specimens harboring *ALK* fusion, *EGFR* mutation, *KRAS* mutation, or none (Table 2). ILS31007 had *EML4-ALK* variant 3a, and markedly expressed mRNA, protein, and phosphorylation of C-terminal ALK compared with the other three *ALK* fusion–negative tissue specimens (Fig. 3a, b and Additional file 9: Figures S5a, b, c and Additional file 10: Figure S6). Expression of C-terminal ALK protein was not detected in the other three *ALK* fusion–negative tissues (Fig. 3b). Meanwhile, the N-terminal EML4 protein was expressed in all tumor tissues independent of *ALK* fusion status (Fig. 3b and Additional file 8: Figure S4b).

Then, we used the non-tumor NAT specimens to investigate whether transcription of *EML4* is constitutively activated even in normal lung cells. Firstly, in *ALK* fusion–positive ILS31007, we confirmed that the NAT specimens did not include any tumor cells (Additional file 11: Figure S7) and that the *EML4* mRNA levels at exon1/2, 2/3, 5/6, or 6/7 in NAT were almost the same as those in the tumor tissues (Fig. 3c and Additional file 12: Figure S8). In the other *ALK* fusion–negative NAT specimens, more *EML4* mRNA was expressed at each exon than in the tumor tissues. These data suggest that *EML4* is constitutively transcribed by its own strong promoter activity in not only *ALK* fusion–positive and –negative lung cancer cells but also in non-tumor lung cells.

### Expression of RET in cancer cell lines with or without *RET* fusion

*RET* is fused with genes such as *KIF5B* or *CCDC6* in approximately 1% of lung cancers [2]. We therefore examined RET expression at the C-terminus in 37 lung cancer cell lines with or without *RET* fusion (Table 1). The LC-2/ad cell line contained *CCDC6-RET* fusion (Additional file 5: Figure S1b and Additional file 6: Figure S2). One of the ALK-TKIs, alectinib, which has the potential to inhibit RET kinase activity [16], suppressed the cell growth of the LC-2/ad cell (Additional file 7: Table S5). In the LC-2/ad cell, the mRNA level of the C-terminal RET region, which is backward from the breakpoint at exon 12 in *RET* rearrangement [14], was elevated by at least 300-fold more than the median of all 37 cell lines, and protein expression at the C-terminus was also high (Figs. 1b and 2). Interestingly, despite being a *RET* fusion–negative cell line, the Calu-6 cell markedly expressed *RET* mRNA from the N- to the C-terminal region and C-terminal RET protein, and the cell was insensitive to alectinib (Figs. 1b and 2, Additional file 7: Table S5 and Additional file 13: Figure S9b). None of the 35 cell lines other than LC-2/ad and Calu-6 expressed any mRNA or C-terminal RET protein. Regardless of RET status, the protein expression of CCDC6 and KIF5B was detected in all 37 cell lines (Figs. 1b, 2 and Additional file 8: Figure S4c).

### Expression of ROS1 in cancer cell lines with or without *ROS1* fusion

*ROS1* is rearranged with genes such as *SCL34A2* or *CD73* in approximately 1% of lung cancers [2]. We therefore examined ROS1 expression at the C-terminus in 37 lung cancer cell lines with or without *ROS1* fusion (Table 1). The HCC78 cell line contained *SLC34A2-ROS1* fusion (Additional file 5: Figure S1c and Additional file 6: Figure S2). The ROS1-TKIs, crizotinib and ceritinib, suppressed cell growth (Additional file 7: Table S5). In HCC78 cells, the mRNA level of the C-terminal ROS1 region, which is backward from the breakpoint at exon 32 or 34 in *ROS1* rearrangement [17], was elevated by at least 2000-fold more than the median of all 37 cell lines, and high protein expression at the C-terminus was detected (Figs. 1c and 2).

## Discussion

Chromosomal rearrangements involving *ALK*, *RET*, and *ROS1* are attractive anticancer targets that provide opportunities for therapies for patients with NSCLC [9]. As described in previous preclinical studies [18, 19], only the three *ALK* fusion–positive cell lines in the 12 cell lines tested were sensitive to all ALK-TKIs (alectinib, crizotinib and ceritinib) through the suppression of phosphorylation of ALK signaling pathways involving STAT3/AKT/ERK (Additional file 7: Table S5, Additional file 14: Figure S3, Additional file 13: Figure S9, Additional file 15: Figure S10, and Additional file 16: Figure S11), indicating that the growth of these *ALK* fusion–positive cell lines strongly depends on the signal from ALK. In the US, both a FISH test using the Vysis ALK break apart FISH probe kit (Abbott Laboratories) and an IHC test using the Ventana ALK (D5F3) CDx assay (Ventana Medical Systems; Tucson, AZ) have been approved as a companion diagnostic (CDx) test for crizotinib [20]. In Japan, the Vysis FISH test has been approved as a CDx test for crizotinib, and a diagnostic tool combining the Vysis FISH test with an IHC test using the N-Histofine ALK Detection kit (Nichirei Biosciences) has been approved as a CDx test for alectinib [6]. These two IHC kits, which respectively include clone D5F3 or 5A4 as a primary antibody directed to C-terminal ALK protein, were highly concordant with the ALK FISH tests. However, it was reported that IHC-positive and FISH-negative patients were occasionally present, with these discordant patients showing a clinical response to crizotinib [6, 21, 22]. To identify patients who would be suitable for ALK-TKIs, the accurate diagnosis of *ALK* fusion is a critical issue. In this study, we focused on the reliability of the detection of C-terminus ALK protein for the diagnosis of *ALK* fusion using various types of lung cancer cell lines and tissues. We found that the promoter of *EML4* was constitutively activated in lung cancer as well as normal cells independent of *ALK* fusion, and C-terminal ALK protein level and phosphorylation were specifically elevated in *ALK* fusion–positive cancer cells (Figs. 2 and 3b). As previously demonstrated [4, 7], these findings suggest that wild-type ALK is silenced in normal lung cells because of lack of production, but when C-terminal *ALK* is fused to N-terminal *EML4* in normal cells, the transcription of the kinase domain of ALK is activated by the constant promoter activity of *EML4*, and the resultant abundantly produced EML4-fused ALK leads to cancer through aberrant ALK signal transduction. Therefore, IHC tests for ALK, such as those using the Ventana and N-Histofine kits, could be sufficiently reliable diagnostic methods in the treatment of patients with lung cancer using ALK-TKIs.

As previously described in preclinical studies [16, 23, 24], 2 cell lines tested only LC-2/ad or HCC78 cell line with *RET* or *ROS1* fusion were sensitive to RET-TKI (alectinib) or ROS1-TKIs (crizotinib and ceritinib) by suppressing the level of phosphorylation of STAT3/AKT/ERK, which are located downstream of RET or ROS1 kinase, respectively (Additional file 7: Table S5, Additional file 13: Figure S9a, Additional file 15: Figure S10 and Additional file 16: Figure S11). No IHC or FISH CDx tests that detect *RET* or *ROS1* fusions have been approved, but an IHC test using an antibody clone, EPR2871 (Abcam), is under investigation [10]. Although RET expression was low in normal lung tissue [25], discordant results between the IHC test and the FISH test for RET have been reported [26, 27]. In this study, the promoter activity of *KIF5B* or *CCDC6*, which are two major genes involved in fusion with C-terminal RET, was constitutively activated in every cell line, and the expression of C-terminal RET was considerably high not only in the *RET* fusion–positive cell line LC-2/ad, but also in one of the five *RET* fusion–negative and *KRAS*-mutated cell lines Calu-6. Wild-type RET expression in Calu-6 cells has been also reported by Zhou et al. [28]. The weight of RET protein in LC-2/ad or Calu-6 cells was, respectively, 50 to 100 kDa or 100 to 200 kDa (Figs. 1b and 2), which is approximately the same weight as that reported previously for, respectively, CCDC6-fused RET or wild-type RET [29, 30]. Just as surprisingly, in 37 cell lines, phosphorylation of the C-terminal RET domain was only detected in the *RET* fusion–negative and *MET*-amplified NCI-H1993 cell line (Fig. 2), but that elevation of the level of MET phosphorylation by trans-phosphorylation of MET within RET and MET heterodimers was reported in NCI-H1993 cells [31]. Both Calu-6 and NCI-H1993 cells were completely insensitive to alectinib (Additional file 7: Table S5 and Additional file 13: Figure S9b and c), which means that cell growth with wild-type RET is independent of RET kinase even if the cells have high expression or high phosphorylation of RET, and RET kinase would be an oncogenic growth driver after fusion with, for example, *CCDC6* or *KIF5B*. Taking all these findings together, the stand-alone RET IHC test for the detection of C-terminal RET protein may cause misleading judgments of *RET* fusion, and CDx tests with both IHC and FISH or RT-PCR would be needed in the treatment of patients with *RET* fusion–positive lung cancer using RET-TKIs.

Regarding the ROS1 IHC test using an antibody for the C-terminus, some patients were reported to show discordant results between IHC and FISH tests despite low expression of ROS1 protein in normal lung tissue [11, 32]. However, in this study, the *ROS1* fusion–positive cell line, HCC78, harboring an *SLC34A2-ROS1* fusion only showed protein expression at the C-terminal domain and a sensitivity to the ROS1-TKIs by inhibiting ROS1 signaling pathways involving STAT3/AKT/ERK (Figs. 1c and 2 and Additional file 7: Table S5 and Additional file 15: Figure S10). *SLC34A2* and *CD74* are two genes fused to C-terminal *ROS1*, and *SLC34A2* mRNA expression was shown in tissues and cell lines of NSCLC as well as normal lung tissues [33–35]. CD74 protein was also strongly expressed in many lung cancer tissues [36]. Therefore, the C-terminal ROS1 protein level could only be elevated by the strong promoter activity of genes such as *SLC34A2* or *CD74* in *ROS1* fusion–positive lung cancer cells, which suggests that the ROS1 IHC test is a reliable diagnostic test for the detection of patients with lung cancer who have *ROS1* fusion.

On the other hand, ALK or ROS1 IHC tests occasionally showed positive results even in patient samples diagnosed by FISH or RT-PCR tests to be fusion-negative [6, 8, 11]. As one of the causes of this IHC+/FISH- discordance, it was reported that wild-type FISH signals in fusion–positive cases were caused by rare atypical chromosomal rearrangements with *EML4* and *ALK* [6]. In addition, Takeuchi K et al. showed that ALK expression is detected in some *ALK* fusion–negative cases with small-cell carcinoma, large-cell neuroendocrine carcinoma, and poorly differentiated carcinoma [7]. Hyper-methylation of promoter and copy number gain of *ROS1* were reported as one of the mechanisms that activate ROS1 expression in fusion-negative carcinomas [37]. However, the possible factors of discordances mentioned above have not been fully clarified at present. This study with 37 lung cancer cell lines and four tissues did not reproduce the phenomenon of discordance. Therefore, further studies using a larger panel with various types of lung cancer cell lines and tissues would be useful to elucidate the causes of discordance in clinical ALK or ROS1 IHC tests.

Next-generation sequencing (NGS) technology enables high-throughput and multiplex analysis of various driver oncogenes. For NSCLC, NGS-based tumor-profiling multiplex gene panels, such as Oncomine Dx target test or FoundationOne CDx, have recently been approved as companion diagnostics to detect mutations of EGFR and BRAF, or fusions of ALK and ROS1 in the US [38]. These NGS panels are also designed to detect *RET* fusions [39, 40]. At present, clinical diagnosis to select patients with ALK fusion–positive NSCLC is predominantly performed by IHC test, while NGS screening might have the potential to test for multiple gene alterations in a quick single analysis. In this study, we could not compare the analysis of fusions by IHC with that by NGS since we have no data on NGS. However, evaluations of the usability of diagnosis by NGS compared to IHC or FISH in NSCLC specimens showed that NGS screenings could provide an alternative method of detecting fusion genes to IHC or FISH tests [39–41]. Therefore, further studies of NGS in addition to C-terminal protein expression analysis using NSCLC cell lines would be a strong support to precise selection of NSCLC patients with fusion genes by NGS with or without IHC.

## Conclusion

We demonstrated that the transcription levels of *ALK*- or *RET*-fusion partner genes, such as *EML4*, *CCDC6* and *KIF5B*, were constitutively activated in lung cancer cells, and the expression at the C-terminal region of ALK, RET, or ROS1 was also markedly elevated in each fusion-positive lung cancer cell. Moreover, although the expression of ALK and ROS1 at the C-terminus was very limited in all fusion-negative cancer cells, the expression or phosphorylation of C-terminal RET was markedly elevated in 2 of the 36 *RET* fusion–negative cancer cells. Our findings suggest that the measurement of C-terminal ALK or ROS1 protein could be a reliable diagnostic method for each fusion, whereas measuring C-terminal RET protein might be a diagnostic method with a potential to give false-positive results when detecting *RET* fusion in patients with lung cancer.

## Additional files

Additional file 1: **Table S1.** Taqman probes for fusion (DOCX 26 kb)

Additional file 2: **Table S2.** Taqman probes for exons of mRNA (DOCX 28 kb)

Additional file 3: **Table S3.** Target terminus of antibody (DOCX 28 kb)

Additional file 4: **Table S4.** Exon count of mRNA (DOCX 26 kb)

Additional file 5: **Figure S1.** RT-PCR analysis of ALK, RET, or ROS1 fusion in 37 cancer cell lines. The mRNA expression of variant 1, 2, 3a, or 3b of EML4-ALK (a), KIF5B-RET, CCDC6-RET (b) or SLC34A2-ROS1 (c) was determined by RT-PCR using the Taqman probes shown in Additional file 1: Table S1. Red lines show fusion gene–positive cell lines and green lines show fusion gene–negative cell lines (PPTX 129 kb)

Additional file 6: **Figure S2.** Summary of ALK, RET, or ROS1 fusion in 37 cancer cell lines. Plot of normalized values calculated from the data in Additional file 5: Figure S1 (PPTX 50 kb)

Additional file 7: **Table S5.** IC_50_s of ALK-TKIs and erlotinib (DOCX 28 kb)

Additional file 8 **Figure S4.** Comparison of molecular weights between wild-type and fusion protein. After high exposure of N-terminal EML4 in the results shown in Fig. 2, two sizes of EML4 protein were detected in NCI-H2228 and SNU-2292 cell lines (left-hand figure), and we assumed that the larger protein was wild-type EML4 (arrow) and the smaller protein was ALK-fused EML4 (arrowhead), after referring to the protein weight of EML4-ALK detected by an ALK C-terminus antibody (right-hand figure) (a). In the same way, we assumed that the larger and smaller proteins were respectively wild-type EML4 (arrow) and ALK-fused EML4 (arrowhead) in the results for the ILS31007 tumor tissue specimen shown in Fig. 3b (b), and the larger and smaller proteins were respectively wild-type CCDC6 (arrow) and RET-fused CCDC6 (arrowhead) in the results for CCDC6 in the LC-2/ad cell line shown in Fig. 2 (c) (PPTX 600 kb)

Additional file 9: **Figure S5.** RT-PCR analysis of ALK, RET, or ROS1 fusion in 4 tumor tissues. The mRNA expression of variant 1, 2, 3a, or 3b of EML4-ALK (a), KIF5B-RET, CCDC6-RET (b) or SLC34A2-ROS1 (c) was determined by RT-PCR using the Taqman probes shown in Additional file 1: Table S1. Red lines show fusion gene–positive tumor tissue, and green lines show fusion gene–negative tumor tissue (PPTX 122 kb)

Additional file 10: **Figure S6.** Summary of ALK, RET, or ROS1 fusion in 4 tumor tissue specimens. Plot of normalized values calculated from the data in Additional file 9: Figure S5 (PPTX 45 kb)

Additional file 11: **Figure S7.** Analysis by IHC and FISH of ALK in ILS31007 tissue specimen. The FFPE specimens with tumor tissue (upper panel) and the paired NAT specimens (lower panel) were obtained from BioreclamationIVT. IHC (left panel) and FISH (right panel) analyses for ALK rearrangement were performed with, respectively, the N-Histofine ALK detection kit (Nichirei Biosciences; Tokyo, Japan) and the Vysis ALK break apart FISH probe kit (Abbott laboratories; Abbott Park, IL) at a commercial clinical laboratory, LSI Medience (Tokyo, Japan). Images were captured using standard settings by the BZ900 (Keyence; Osaka, Japan) (PPTX 754 kb)

Additional file 12: **Figure S8.** RT-PCR analysis of EML4 in four tissue specimens. The relative EML4 mRNA expression at each exon in NAT specimens was calculated as the ratio of the normalized values with GAPDH mRNA to those in tumor tissues. Each bar represents the mean + SD (*n* = 3) (PPTX 45 kb)

Additional file 13: **Figure S9.** Western blotting analysis in three cancer cell lines with or without RET fusion. Cell lysates were harvested after 2 h of treatment with each drug at the concentrations shown (nM). The levels of RET phosphorylation in LC-2/ad were undetectable by this western blotting system (PPTX 2307 kb)

Additional file 14: **Figure S3.** Western blotting analysis in 3 cancer cell lines with ALK fusion. Cell lysates were harvested after 2 h of treatment with each drug at the concentration shown (nM). The antibodies were obtained from Cell signaling technology. (PPTX 2483 kb)

Additional file 15: **Figure S10.** Western blotting analysis in a cancer cell line with ROS1 fusion. Cell lysates were harvested after 2 h of treatment with each drug at the concentrations shown (nM) (PPTX 797 kb)

Additional file 16: **Figure S11.** Western blotting analysis in five cancer cell lines without any fusion gene. Cell lysates were harvested after 2 h of treatment with each drug at the concentrations shown (nM) (PPTX 2123 kb)

## Abbreviations

*ALK*: Anaplastic lymphoma kinase
*BRAF*: v-Raf murine sarcoma viral oncogene homolog B1
*CCDC6*: Coiled-coil domain containing 6
CDx: Companion diagnostics
COSMIC: Catalogue of somatic mutations in cancer
C-terminal: Carboxy-terminal
*EGFR*: Epidermal growth factor receptor
*EML4*: Echinoderm microtubule associated protein like 4
FISH: Fluorescence in situ hybridization
*HER-2*: Human epidermal growth factor receptor 2
IHC: Immunohistochemistry
*KIF5B*: Kinesin family member 5
*KRAS*: v-Ki-ras2 kirsten rat sarcoma viral oncogene homolog
*MET*: MET proto-oncogene
NAT: Non-tumor normal adjacent tissue
*NRAS*: Neuroblastoma RAS viral (v-ras) oncogene homolog
NSCLC: Non-small cell lung cancer
N-terminal: Amino-terminal
*PIK3CA*: Phosphoinositide-3-kinase, catalytic, α polypeptide
*RET*: RET proto-oncogene
*ROS1*: v-Ros UR2 sarcoma virus oncogene homolog 1
RT-PCR: Reverse transcription polymerase chain reaction
*SLC34A2*: Solute carrier family 34 member 2
TKI: Tyrosine kinase inhibitor
