# Supplementary material for: Expression of C-terminal ALK, RET, or ROS1 in lung cancer cells with or without fusion
Source: BMC Cancer. 2019 Apr 3;19:301. doi: 10.1186/s12885-019-5527-2 (PMC6446279; doi:10.1186/s12885-019-5527-2)
Supplement: Supplementary file 1 — Table S1. Taqman probes for fusion (DOCX 26 kb) [file 12885_2019_5527_MOESM1_ESM.docx]

**Table S1**

| Probe ID | Target mRNA |
| --- | --- |
| Hs04397012 | *EML4-ALK* variant 1 |
| Hs04397036 | *EML4-ALK* variant 2 |
| Hs04397070 | *EML4-ALK* variant 3a |
| Hs04396582 | *EML4-ALK* variant 3b |
| Hs04396863 | *KIF5B-RET* |
| Hs04421293 | *CCDC6-RET* |
| Hs04396942 | *SLC34A2-ROS1* |
| 4352934E | *GAPDH* |
