# Supplementary material for: Expression of C-terminal ALK, RET, or ROS1 in lung cancer cells with or without fusion
Source: BMC Cancer. 2019 Apr 3;19:301. doi: 10.1186/s12885-019-5527-2 (PMC6446279; doi:10.1186/s12885-019-5527-2)
Supplement: Supplementary file 2 — Table S2. Taqman probes for exons of mRNA (DOCX 28 kb) [file 12885_2019_5527_MOESM2_ESM.docx]

**Table S2**

| Probe ID | Target exon of mRNA |
| --- | --- |
| Hs01040671 | *EML4* junction exon 1 to 2 |
| Hs01040675 | *EML4* junction exon 2 to 3 |
| Hs01040678 | *EML4* junction exon 5 to 6 |
| Hs01040679 | *EML4* junction exon 6 to 7 |
| Hs00608295 | *ALK* junction exon 3 to 4 |
| Hs01058323 | *ALK* junction exon 9 to 10 |
| Hs01058315 | *ALK* junction exon 18 to 19 |
| Hs01058317 | *ALK* junction exon 22 to 23 |
| Hs00608289 | *ALK* junction exon 24 to 25 |
| Hs00608292 | *ALK* junction exon 27 to 28 |
| Hs01120027 | *RET* junction exon 2 to 3 |
| Hs01120030 | *RET* junction exon 5 to 6 |
| Hs01120021 | *RET* junction exon 11 to 12 |
| Hs04259656 | *RET* exon 19 |
| Hs04259657 | *RET* exon 20 |
| Hs01090592 | *ROS1* junction exon 11 to 12 |
| Hs00177228 | *ROS1* junction exon 21 to 22 |
| Hs01090613 | *ROS1* junction exon 30 to 31 |
| Hs01090617 | *ROS1* junction exon 34 to 35 |
| Hs01090625 | *ROS1* junction exon 41 to 42 |
| Hs01090626 | *ROS1* junction exon 42 to 43 |

**Table footnote**

The exon number of each probe is referred from datasheet of supplier and NCBI reference sequence database (RefSeq). Other than Hs04259656 and Hs04259657, each probe spans an exon junction and was selected not to detect any contaminated DNA. Both Hs04259656 and Hs04259657, which span the same exon, were selected because there is no validated probe to detect an exon junction around exon 19 to 20 of *RET* mRNA.
