# Supplementary material for: Expression of C-terminal ALK, RET, or ROS1 in lung cancer cells with or without fusion
Source: BMC Cancer. 2019 Apr 3;19:301. doi: 10.1186/s12885-019-5527-2 (PMC6446279; doi:10.1186/s12885-019-5527-2)
Supplement: Supplementary file 3 — Table S3. Target terminus of antibody (DOCX 28 kb) [file 12885_2019_5527_MOESM3_ESM.docx]

**Table S3**

| Antibody | Supplier | Code number^1^ | Target site^1^ | Target terminus^2^ |
| --- | --- | --- | --- | --- |
| EML4 | Cell Signaling | 12156 | S188 in exon 5 | N-terminus |
| Phosphorylation of ALK | Cell Signaling | 9687 | Phosho-Y1282/1283 in exon 26 | C-terminus |
| ALK | Cell Signaling | 3633 | C-terminus | C-terminus |
| KIF5B | Abcam | ab167429 | Amino-acids 1 to 100 in exon 1 to 4 | N-terminus |
| CCDC6 | Santa Cruz | sc-100309 | ND | Unknown |
| Phosphorylation of RET | Cell Signaling | 3221 | Phosho-Y905 in exon 15 | C-terminus |
| RET | Cell Signaling | 14698 | D1000 in exon 18 | C-terminus |
| Phosphorylation of ROS1 | Cell Signaling | 3078 | Phosho-Y2274 in exon 43 | C-terminus |
| ROS1 | Cell Signaling | 3287 | C-terminus | C-terminus |

**Table footnote**

The antibodies of actin and COX4 were obtained from Cell signaling technology (Cell signaling; Danvers, MA). KIF5B and CCDC6 antibodies were Abcam (Cambridge, MA) and Santa cruz biotechnology (Santa cruz; Dallas, TX), respectively. ND: Not demonstrated in supplier’s datasheet. Data was referred from ^1^ datasheet of supplier and ^2^ Ensemble genome browser 86 and Table S4 ^2^.
