# Supplementary material for: Expression of C-terminal ALK, RET, or ROS1 in lung cancer cells with or without fusion
Source: BMC Cancer. 2019 Apr 3;19:301. doi: 10.1186/s12885-019-5527-2 (PMC6446279; doi:10.1186/s12885-019-5527-2)
Supplement: Supplementary file 4 — Table S4. Exon count of mRNA (DOCX 26 kb) [file 12885_2019_5527_MOESM4_ESM.docx]

**Table S4**

| mRNA | RefSeq | Total exons |
| --- | --- | --- |
| *EML4* | NM_019063 | 23 |
| *ALK* | NM_004304 | 29 |
| *KIF5B* | NM_004521 | 26 |
| *CCDC6* | NM_005436 | 9 |
| *RET* | NM_020975 | 20 |
| *ROS1* | NM_002944 | 43 |
