# Supplementary material for: Expression of C-terminal ALK, RET, or ROS1 in lung cancer cells with or without fusion
Source: BMC Cancer. 2019 Apr 3;19:301. doi: 10.1186/s12885-019-5527-2 (PMC6446279; doi:10.1186/s12885-019-5527-2)
Supplement: Supplementary file 5 — Figure S1. RT-PCR analysis of ALK, RET, or ROS1 fusion in 37 cancer cell lines. The mRNA expression of variant 1, 2, 3a, or 3b of EML4-ALK (a), KIF5B-RET, CCDC6-RET (b) or SLC34A2-ROS1 (c) was determined by RT-PCR using the Taqman probes shown in Additional file 1: Table S1. Red lines show fusion gene–positive cell lines and green lines show fusion gene–negative cell lines (PPTX 129 kb) [file 12885_2019_5527_MOESM5_ESM.pptx]

## Slide 1
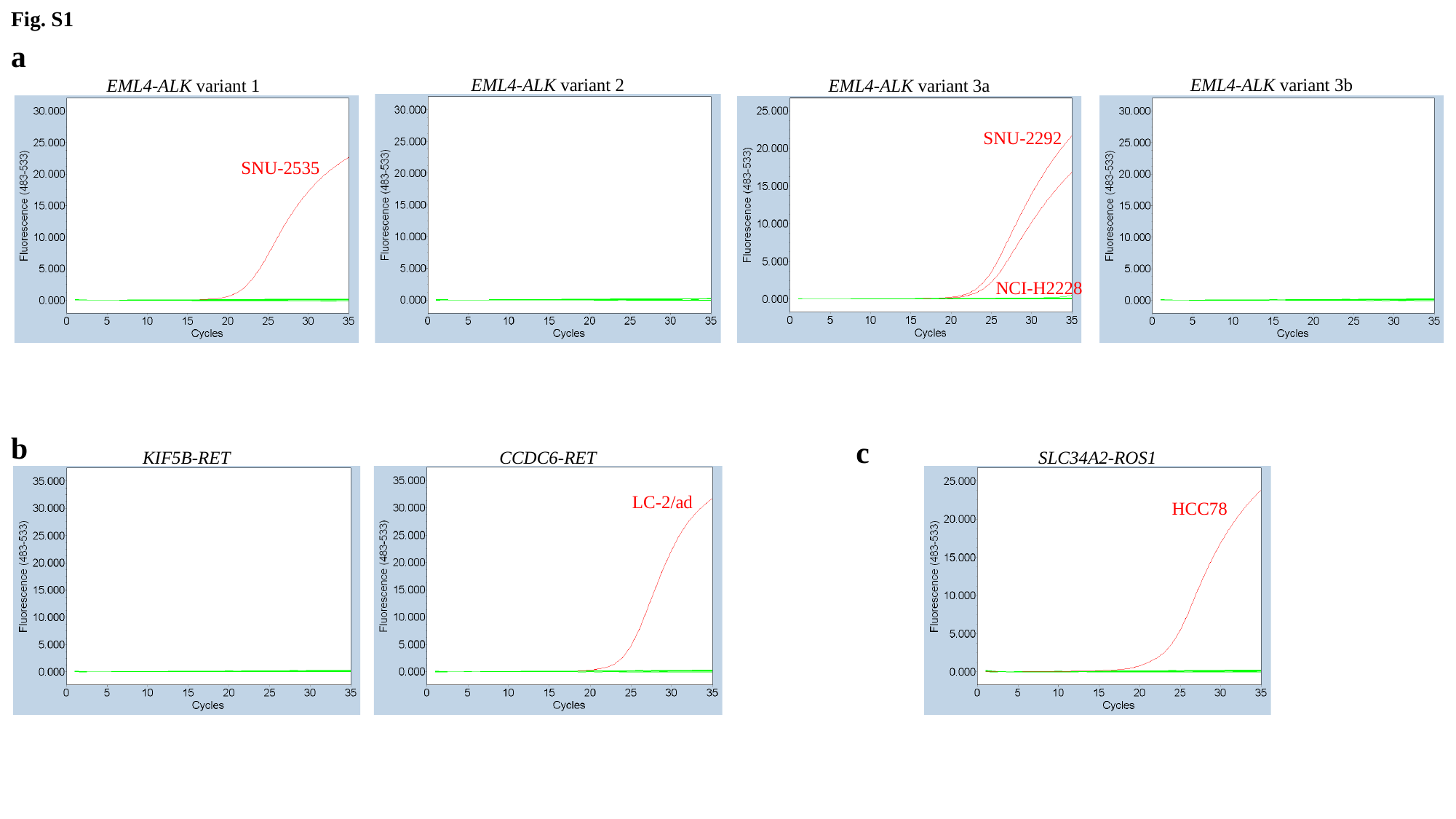

Fig. S1
a
EML4-ALK variant 2
EML4-ALK variant 3b
EML4-ALK variant 1
EML4-ALK variant 3a
SNU-2292
SNU-2535
NCI-H2228
b
c
KIF5B-RET
CCDC6-RET
LC-2/ad
SLC34A2-ROS1
HCC78
