# Supplementary material for: Expression of C-terminal ALK, RET, or ROS1 in lung cancer cells with or without fusion
Source: BMC Cancer. 2019 Apr 3;19:301. doi: 10.1186/s12885-019-5527-2 (PMC6446279; doi:10.1186/s12885-019-5527-2)
Supplement: Supplementary file 7 — Table S5. IC50s of ALK-TKIs and erlotinib (DOCX 28 kb) [file 12885_2019_5527_MOESM7_ESM.docx]

**Table S5**

| Driver gene mutation | Cell line | Alectinib (nM) | Crizotinib (nM) | Ceritinib (nM) | Erlotinib (nM) |
| --- | --- | --- | --- | --- | --- |
| *EML4-ALK* | NCI-H2228 | 7.9 | 35.2 | 8.6 | >1000 |
|  | SNU-2535 | 39.1 | 448.1 | 41.9 | >1000 |
|  | SNU-2292 | 94.1 | 70.7 | 43.8 | >1000 |
| *EGFR* | NCI-H1975 | >1000 | >1000 | >1000 | >1000 |
| *KRAS* | NCI-H358 | >1000 | 807.1 | >1000 | 688.1 |
|  | Calu-6 | >1000 | >1000 | >1000 | >1000 |
| *ERBB2* | NCI-H1781 | >1000 | >1000 | >1000 | >1000 |
| *BRAF* | NCI-H1755 | >1000 | >1000 | >1000 | >1000 |
| *CCDC6-RET* | LC-2/ad | 337.1 | >1000 | >1000 | >1000 |
| *SLC34A2-ROS1* | HCC78 | >1000 | 96.6 | 291.2 | >1000 |
| *MET* | NCI-H1993 | >1000 | 28.7 | >1000 | >1000 |
| ND | NCI-H1703 | >1000 | 877.9 | >1000 | >1000 |

**Table footnote**

Alectinib was synthesized at Chugai, crizotinib was purchased from Selleck Chemicals (Houston, TX), ceritinib was purchased from Active Biochemicals (Maplewood, NJ), and erlotinib was provided by F. Hoffman-La Roche Ltd. (Basel, Switzerland). Each drug was dissolved in dimethyl sulfoxide. Cells were seeded onto 96-well plates and the drugs were added at the indicated concentrations on the following day. After four days, the viability was determined by crystal violet assay, and the concentration of 50% cell growth inhibition (IC_50_) was calculated. ND: Not detected.
