# Supplementary material for: Expression of C-terminal ALK, RET, or ROS1 in lung cancer cells with or without fusion
Source: BMC Cancer. 2019 Apr 3;19:301. doi: 10.1186/s12885-019-5527-2 (PMC6446279; doi:10.1186/s12885-019-5527-2)
Supplement: Supplementary file 8 — Figure S4. Comparison of molecular weights between wild-type and fusion protein. After high exposure of N-terminal EML4 in the results shown in Fig. 2, two sizes of EML4 protein were detected in NCI-H2228 and SNU-2292 cell lines (left-hand figure), and we assumed that the larger protein was wild-type EML4 (arrow) and the smaller protein was ALK-fused EML4 (arrowhead), after referring to the protein weight of EML4-ALK detected by an ALK C-terminus antibody (right-hand figure) (a). In the same way, we assumed that the larger and smaller proteins were respectively wild-type EML4 (arrow) and ALK-fused EML4 (arrowhead) in the results for the ILS31007 tumor tissue specimen shown in Fig. 3b (b), and the larger and smaller proteins were respectively wild-type CCDC6 (arrow) and RET-fused CCDC6 (arrowhead) in the results for CCDC6 in the LC-2/ad cell line shown in Fig. 2 (c) (PPTX 600 kb) [file 12885_2019_5527_MOESM8_ESM.pptx]

## Slide 1
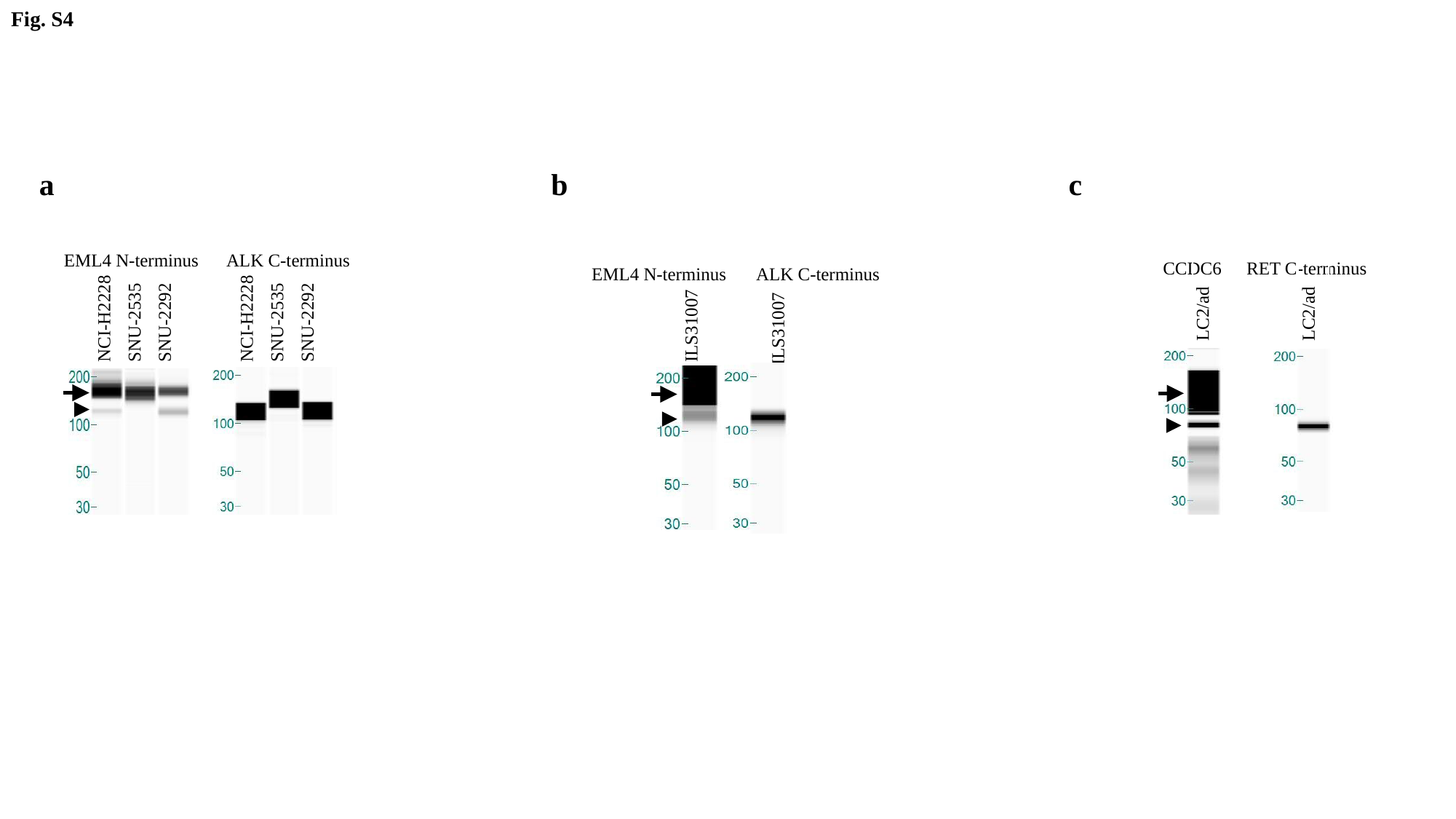

Fig. S4
a
b
c
| LC2/ad |
| --- |
| LC2/ad |
| --- |
EML4 N-terminus
ALK C-terminus
| NCI-H2228 | SNU-2535 | SNU-2292 |
| --- | --- | --- |
| NCI-H2228 | SNU-2535 | SNU-2292 |
| --- | --- | --- |
CCDC6
RET C-terminus
| ILS31007 |
| --- |
| ILS31007 |
| --- |
EML4 N-terminus
ALK C-terminus
