# Supplementary material for: Expression of C-terminal ALK, RET, or ROS1 in lung cancer cells with or without fusion
Source: BMC Cancer. 2019 Apr 3;19:301. doi: 10.1186/s12885-019-5527-2 (PMC6446279; doi:10.1186/s12885-019-5527-2)
Supplement: Supplementary file 9 — Figure S5. RT-PCR analysis of ALK, RET, or ROS1 fusion in 4 tumor tissues. The mRNA expression of variant 1, 2, 3a, or 3b of EML4-ALK (a), KIF5B-RET, CCDC6-RET (b) or SLC34A2-ROS1 (c) was determined by RT-PCR using the Taqman probes shown in Additional file 1: Table S1. Red lines show fusion gene–positive tumor tissue, and green lines show fusion gene–negative tumor tissue (PPTX 122 kb) [file 12885_2019_5527_MOESM9_ESM.pptx]

## Slide 1
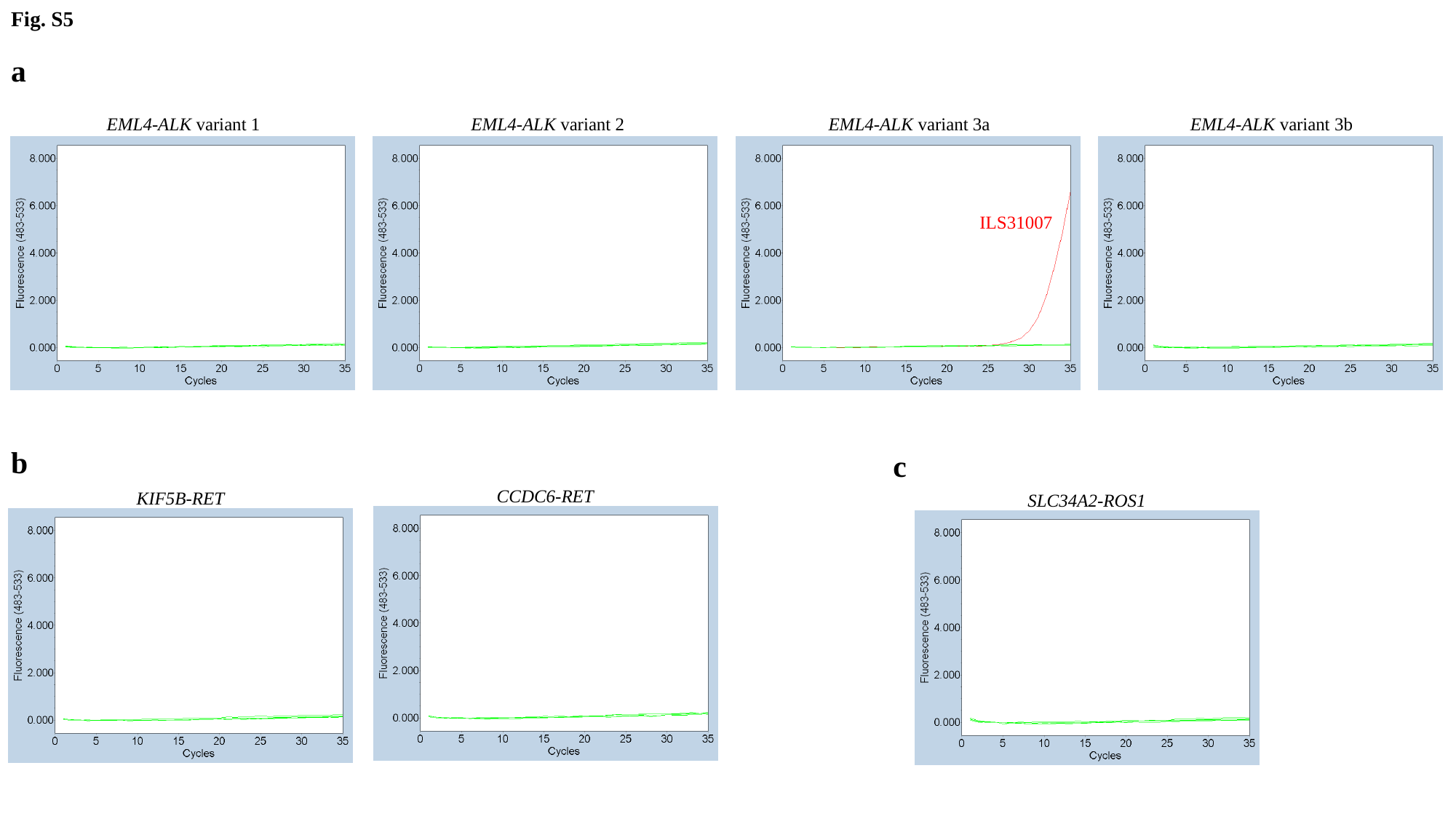

Fig. S5
a
EML4-ALK variant 1
EML4-ALK variant 2
EML4-ALK variant 3a
EML4-ALK variant 3b
ILS31007
b
c
CCDC6-RET
KIF5B-RET
SLC34A2-ROS1
