# Supplementary figures and images for: Expression of C-terminal ALK, RET, or ROS1 in lung cancer cells with or without fusion
Source: BMC Cancer. 2019 Apr 3;19:301. doi: 10.1186/s12885-019-5527-2 (PMC6446279; doi:10.1186/s12885-019-5527-2)

## Slide 1
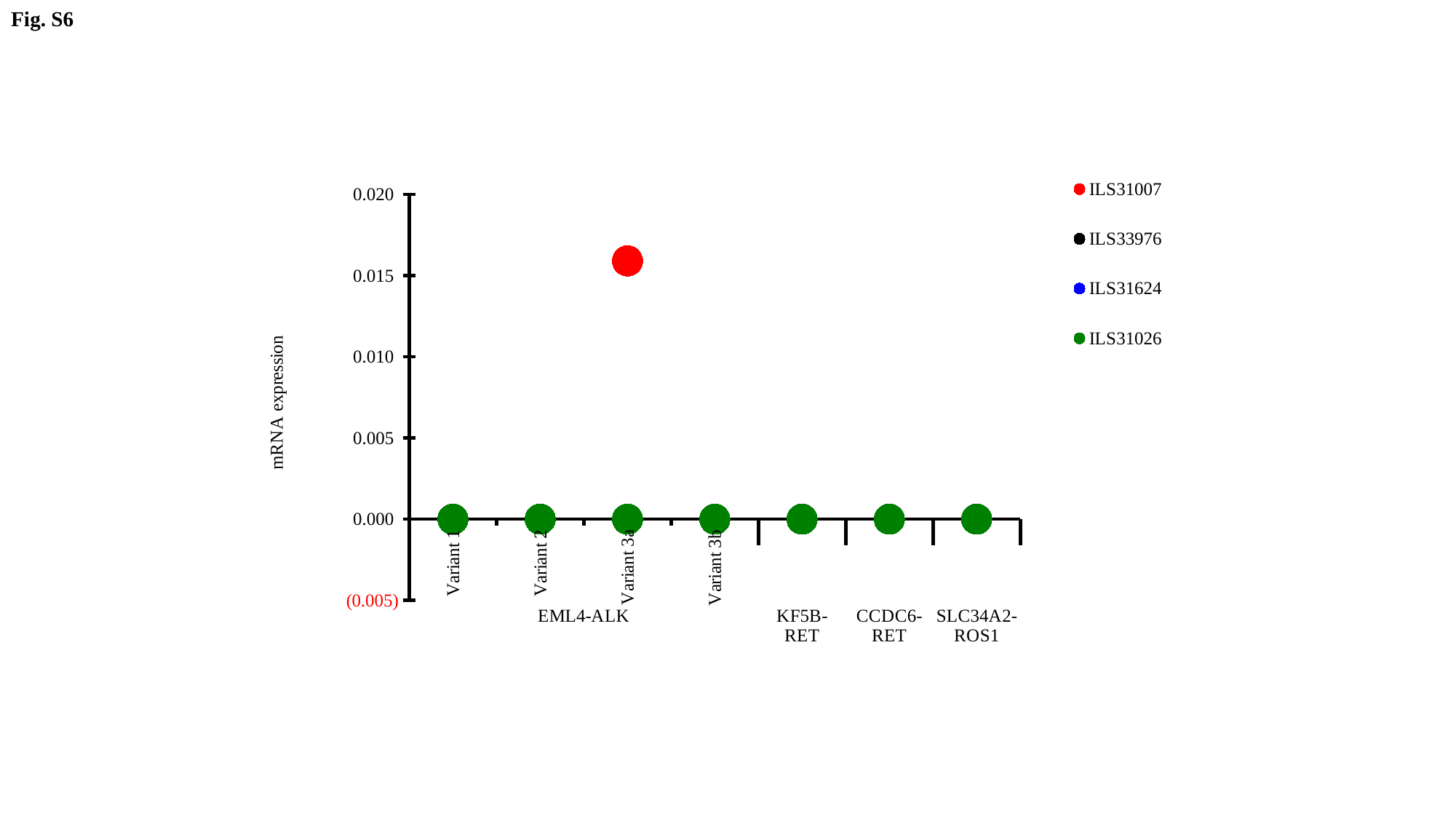

Fig. S6
### Chart
| Category | ILS31007 | ILS33976 | ILS31624 | ILS31026 |
|---|---|---|---|---|
| Variant 1 | 0.0 | 0.0 | 0.0 | 0.0 |
| Variant 2 | 0.0 | 0.0 | 0.0 | 0.0 |
| Variant 3a | 0.0159 | 0.0 | 0.0 | 0.0 |
| Variant 3b | 0.0 | 0.0 | 0.0 | 0.0 |
| | 0.0 | 0.0 | 0.0 | 0.0 |
| | 0.0 | 0.0 | 0.0 | 0.0 |
| | 0.0 | 0.0 | 0.0 | 0.0 |

Supplement: Supplementary file 10 — Figure S6. Summary of ALK, RET, or ROS1 fusion in 4 tumor tissue specimens. Plot of normalized values calculated from the data in Additional file 9: Figure S5 (PPTX 45 kb) [file 12885_2019_5527_MOESM10_ESM.pptx]

## Slide 1
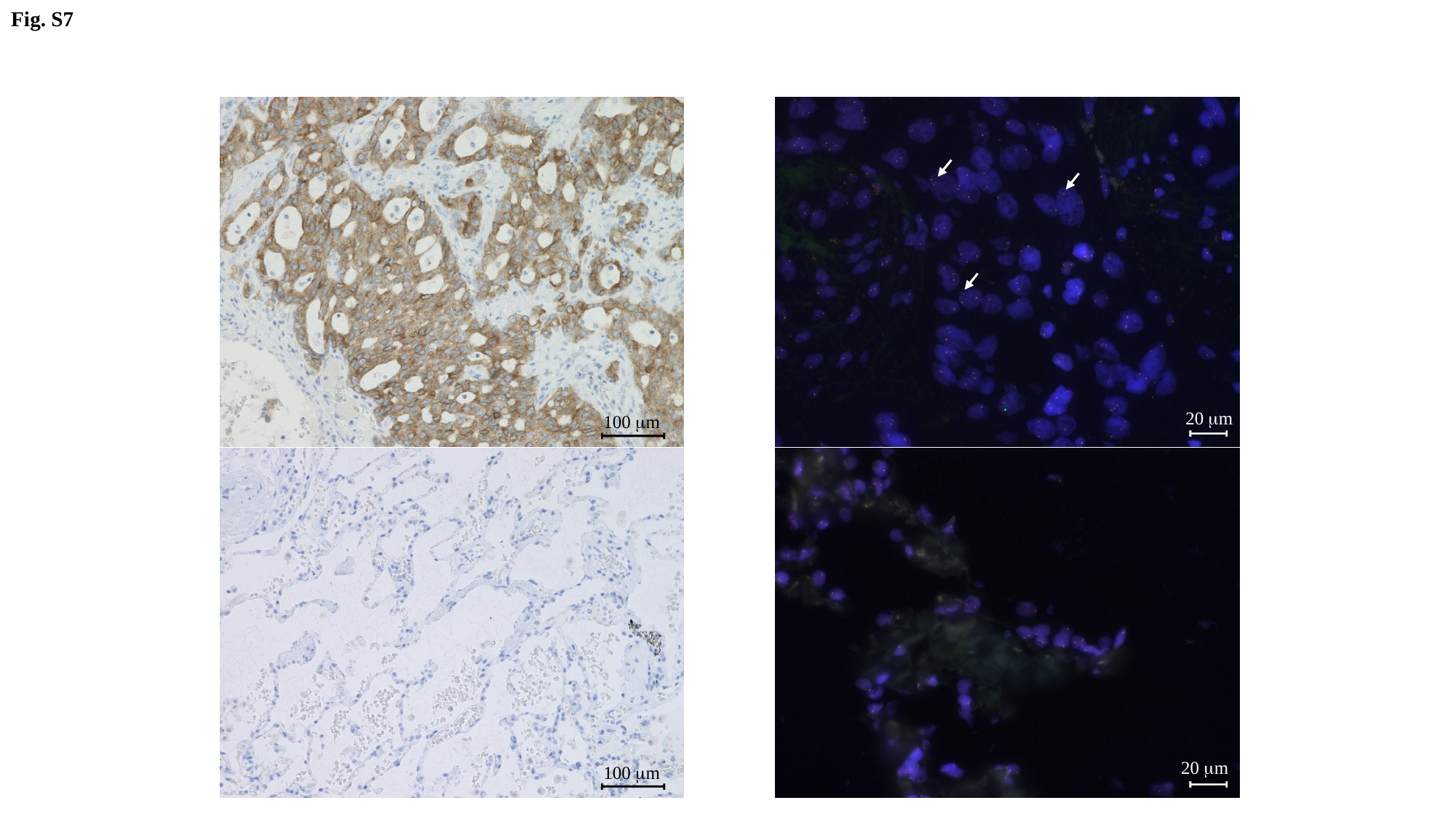

Fig. S7
20 μm
100 μm
20 μm
100 μm

Supplement: Supplementary file 11 — Figure S7. Analysis by IHC and FISH of ALK in ILS31007 tissue specimen. The FFPE specimens with tumor tissue (upper panel) and the paired NAT specimens (lower panel) were obtained from BioreclamationIVT. IHC (left panel) and FISH (right panel) analyses for ALK rearrangement were performed with, respectively, the N-Histofine ALK detection kit (Nichirei Biosciences; Tokyo, Japan) and the Vysis ALK break apart FISH probe kit (Abbott laboratories; Abbott Park, IL) at a commercial clinical laboratory, LSI Medience (Tokyo, Japan). Images were captured using standard settings by the BZ900 (Keyence; Osaka, Japan) (PPTX 754 kb) [file 12885_2019_5527_MOESM11_ESM.pptx]

## Slide 1
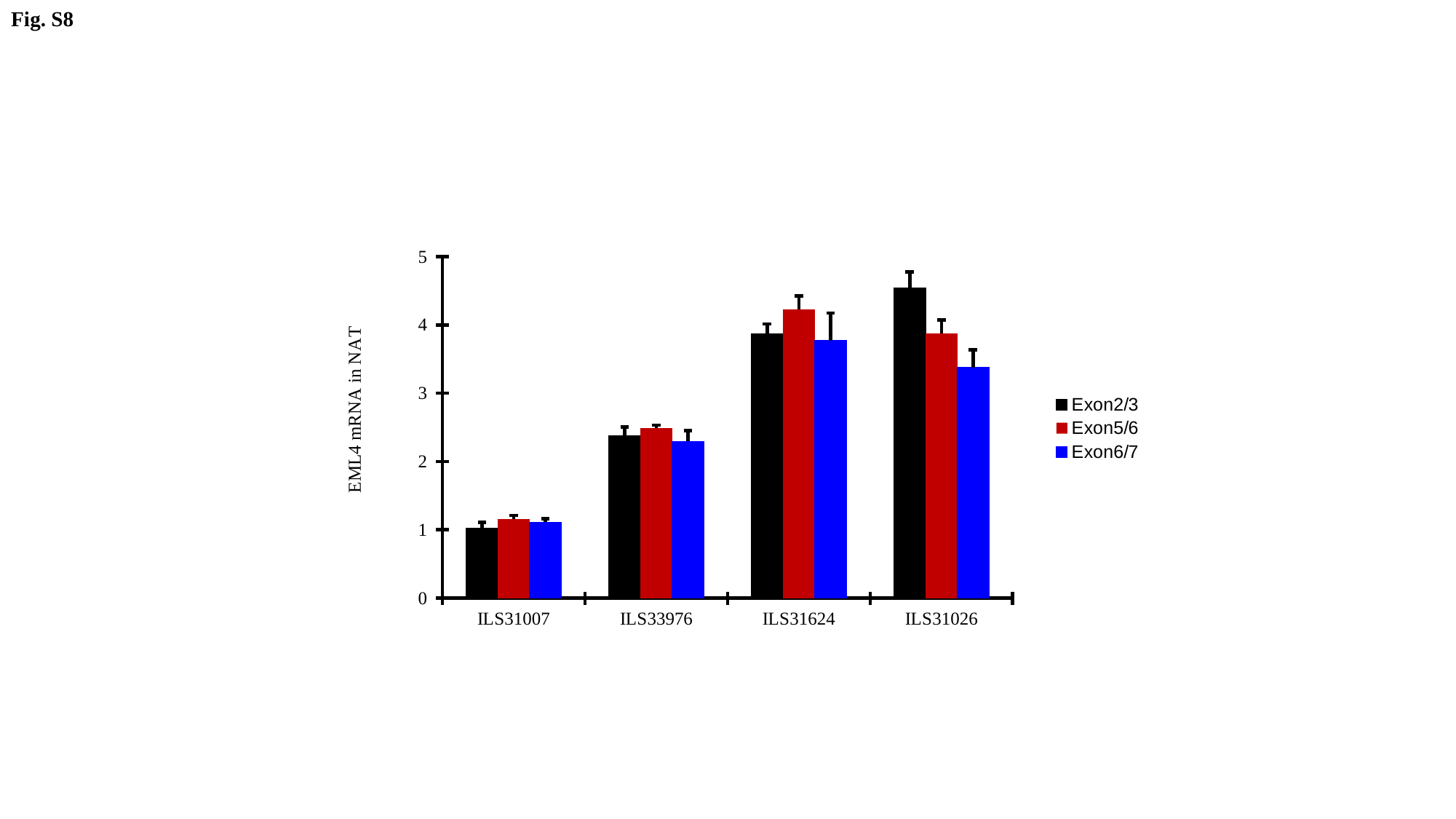

Fig. S8
### Chart
| Category | Exon2/3 | Exon5/6 | Exon6/7 |
|---|---|---|---|
| ILS31007 | 1.026848749095202 | 1.1572879166390928 | 1.1115077437165162 |
| ILS33976 | 2.380706864322317 | 2.4898046647243457 | 2.29685775442514 |
| ILS31624 | 3.865018003951242 | 4.222824544936244 | 3.768962942363943 |
| ILS31026 | 4.535771551346477 | 3.875988513940321 | 3.3791282536734784 |

Supplement: Supplementary file 12 — Figure S8. RT-PCR analysis of EML4 in four tissue specimens. The relative EML4 mRNA expression at each exon in NAT specimens was calculated as the ratio of the normalized values with GAPDH mRNA to those in tumor tissues. Each bar represents the mean + SD (n = 3) (PPTX 45 kb) [file 12885_2019_5527_MOESM12_ESM.pptx]
