# Supplementary material for: Expression of C-terminal ALK, RET, or ROS1 in lung cancer cells with or without fusion
Source: BMC Cancer. 2019 Apr 3;19:301. doi: 10.1186/s12885-019-5527-2 (PMC6446279; doi:10.1186/s12885-019-5527-2)
Supplement: Supplementary file 13 — Figure S9. Western blotting analysis in three cancer cell lines with or without RET fusion. Cell lysates were harvested after 2 h of treatment with each drug at the concentrations shown (nM). The levels of RET phosphorylation in LC-2/ad were undetectable by this western blotting system (PPTX 2307 kb) [file 12885_2019_5527_MOESM13_ESM.pptx]

## Slide 1
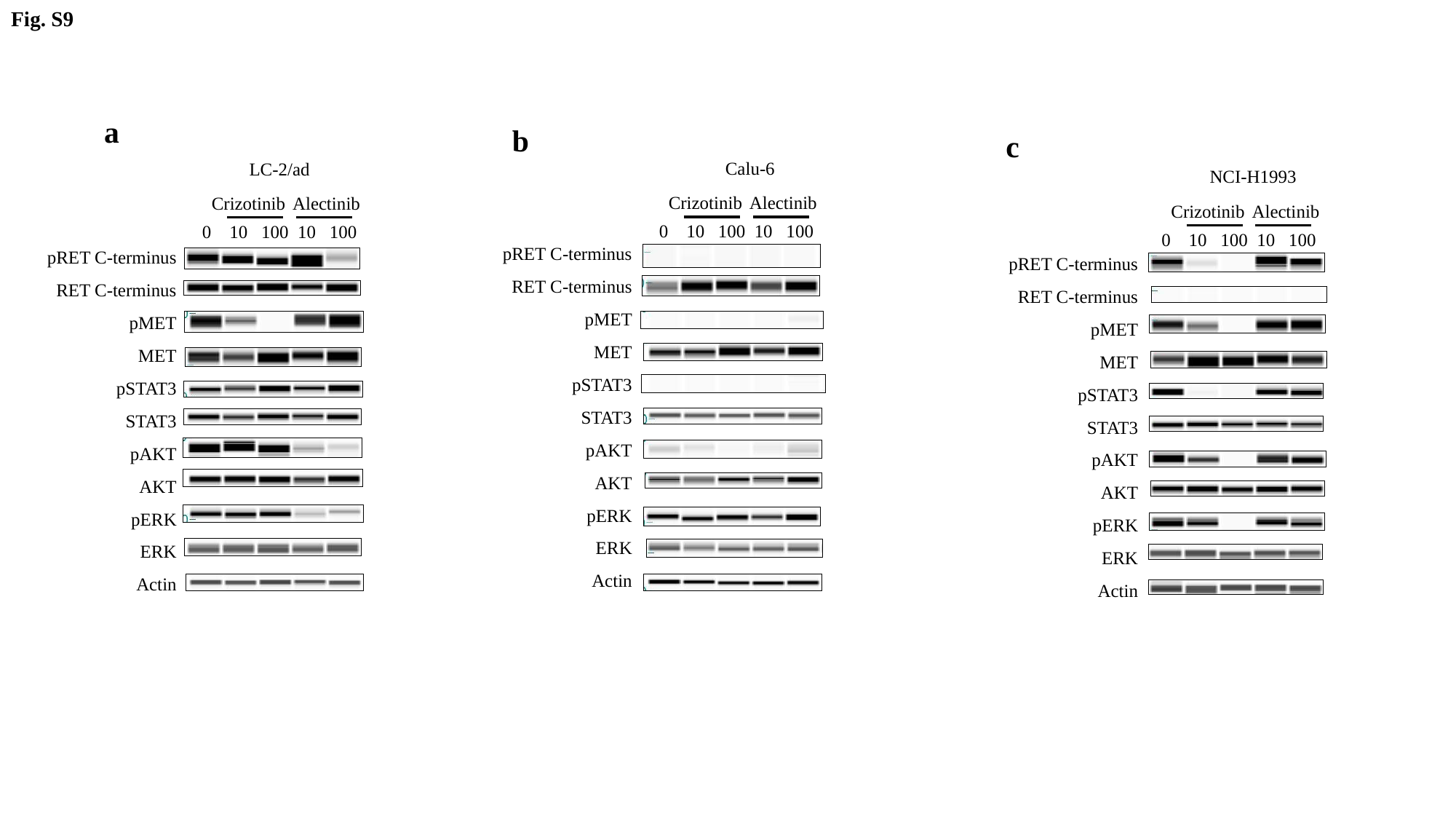

Fig. S9
a
b
c
Calu-6
Crizotinib
Alectinib
0 10 100 10 100
LC-2/ad
Crizotinib
Alectinib
0 10 100 10 100
NCI-H1993
Crizotinib
Alectinib
0 10 100 10 100
pRET C-terminus
RET C-terminus
pMET
MET
pSTAT3
STAT3
pAKT
AKT
pERK
ERK
Actin
pRET C-terminus
RET C-terminus
pMET
MET
pSTAT3
STAT3
pAKT
AKT
pERK
ERK
Actin
pRET C-terminus
RET C-terminus
pMET
MET
pSTAT3
STAT3
pAKT
AKT
pERK
ERK
Actin
