# Supplementary material for: Expression of C-terminal ALK, RET, or ROS1 in lung cancer cells with or without fusion
Source: BMC Cancer. 2019 Apr 3;19:301. doi: 10.1186/s12885-019-5527-2 (PMC6446279; doi:10.1186/s12885-019-5527-2)
Supplement: Supplementary file 14 — Figure S3. Western blotting analysis in 3 cancer cell lines with ALK fusion. Cell lysates were harvested after 2 h of treatment with each drug at the concentration shown (nM). The antibodies were obtained from Cell signaling technology. (PPTX 2483 kb) [file 12885_2019_5527_MOESM14_ESM.pptx]

## Slide 1
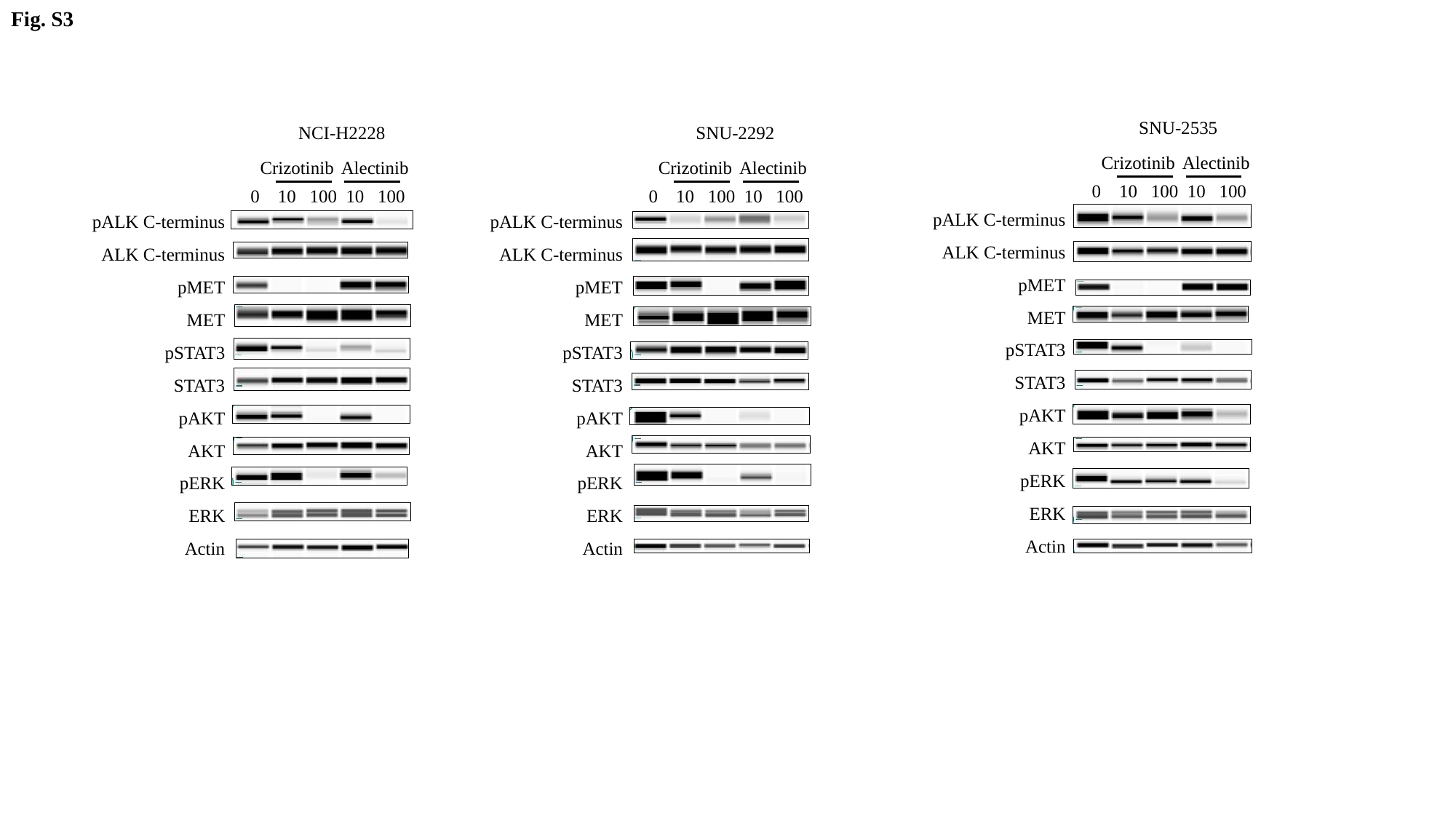

Fig. S3
SNU-2535
Crizotinib
Alectinib
0 10 100 10 100
NCI-H2228
Crizotinib
Alectinib
0 10 100 10 100
SNU-2292
Crizotinib
Alectinib
0 10 100 10 100
pALK C-terminus
ALK C-terminus
pMET
MET
pSTAT3
STAT3
pAKT
AKT
pERK
ERK
Actin
pALK C-terminus
ALK C-terminus
pMET
MET
pSTAT3
STAT3
pAKT
AKT
pERK
ERK
Actin
pALK C-terminus
ALK C-terminus
pMET
MET
pSTAT3
STAT3
pAKT
AKT
pERK
ERK
Actin
