# Supplementary material for: Expression of C-terminal ALK, RET, or ROS1 in lung cancer cells with or without fusion
Source: BMC Cancer. 2019 Apr 3;19:301. doi: 10.1186/s12885-019-5527-2 (PMC6446279; doi:10.1186/s12885-019-5527-2)
Supplement: Supplementary file 15 — Figure S10. Western blotting analysis in a cancer cell line with ROS1 fusion. Cell lysates were harvested after 2 h of treatment with each drug at the concentrations shown (nM) (PPTX 797 kb) [file 12885_2019_5527_MOESM15_ESM.pptx]

## Slide 1
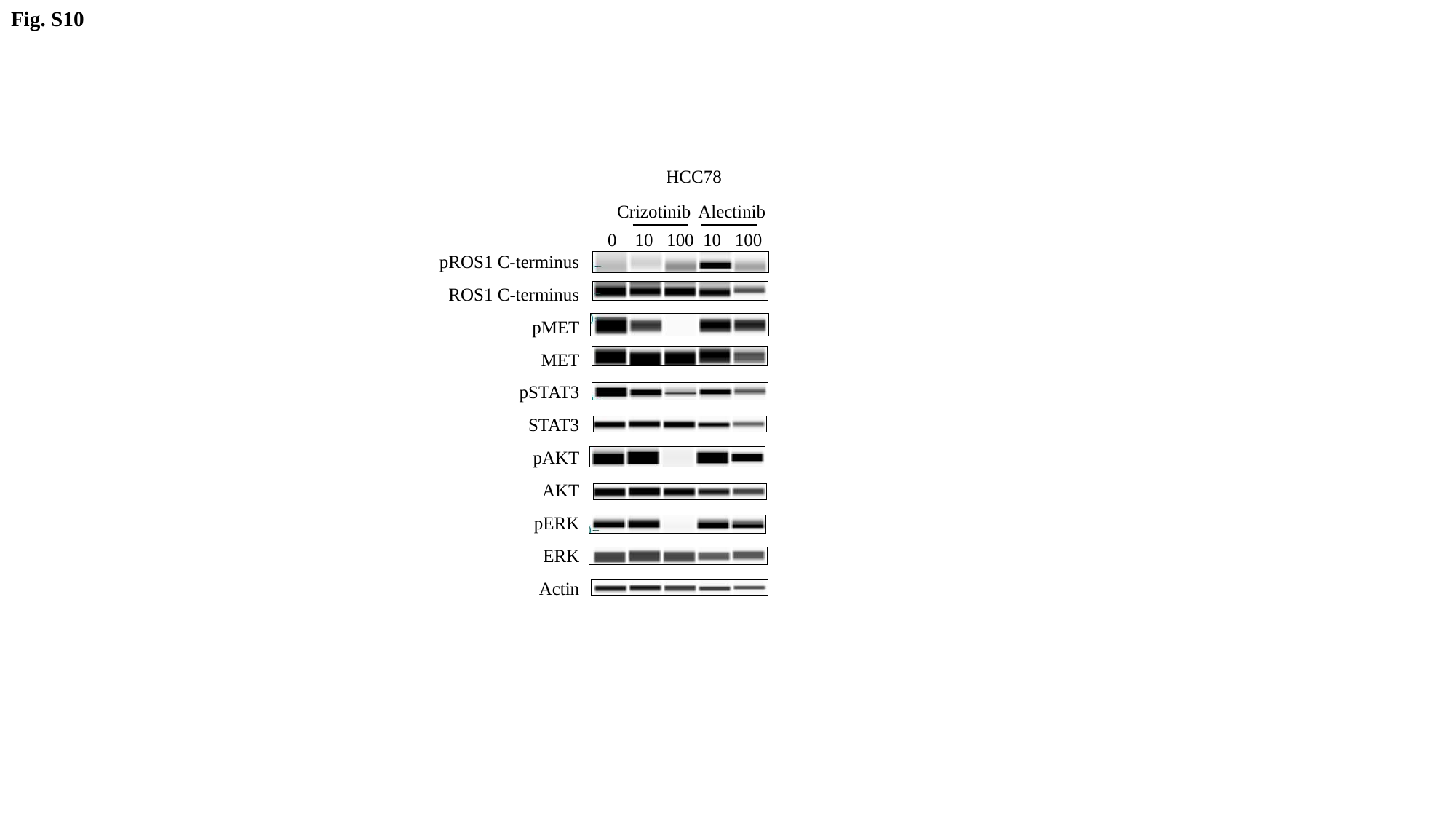

Fig. S10
HCC78
Crizotinib
Alectinib
0 10 100 10 100
pROS1 C-terminus
ROS1 C-terminus
pMET
MET
pSTAT3
STAT3
pAKT
AKT
pERK
ERK
Actin
