# Supplementary material for: Expression of C-terminal ALK, RET, or ROS1 in lung cancer cells with or without fusion
Source: BMC Cancer. 2019 Apr 3;19:301. doi: 10.1186/s12885-019-5527-2 (PMC6446279; doi:10.1186/s12885-019-5527-2)
Supplement: Supplementary file 16 — Figure S11. Western blotting analysis in five cancer cell lines without any fusion gene. Cell lysates were harvested after 2 h of treatment with each drug at the concentrations shown (nM) (PPTX 2123 kb) [file 12885_2019_5527_MOESM16_ESM.pptx]

## Slide 1
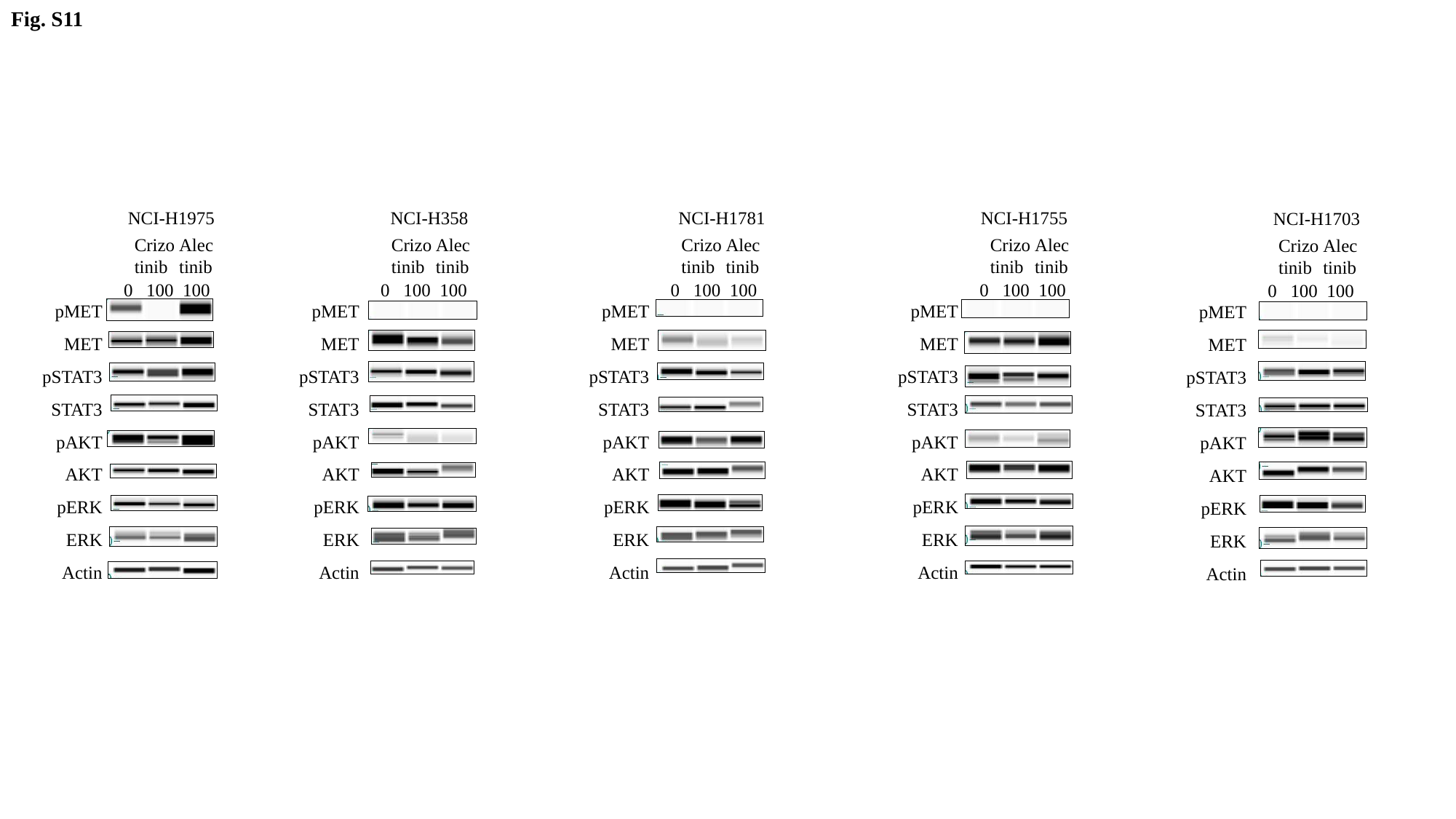

Fig. S11
NCI-H1975
Crizo
tinib
Alec
tinib
0 100 100
pMET
MET
pSTAT3
STAT3
pAKT
AKT
pERK
ERK
Actin
NCI-H358
Crizo
tinib
Alec
tinib
0 100 100
pMET
MET
pSTAT3
STAT3
pAKT
AKT
pERK
ERK
Actin
NCI-H1781
Crizo
tinib
Alec
tinib
0 100 100
pMET
MET
pSTAT3
STAT3
pAKT
AKT
pERK
ERK
Actin
NCI-H1755
Crizo
tinib
Alec
tinib
0 100 100
pMET
MET
pSTAT3
STAT3
pAKT
AKT
pERK
ERK
Actin
NCI-H1703
Crizo
tinib
Alec
tinib
0 100 100
pMET
MET
pSTAT3
STAT3
pAKT
AKT
pERK
ERK
Actin
